# A revised Ladevèze criteria for carbon fiber reinforced laminated plates

**DOI:** 10.1016/j.dib.2019.104498

**Published:** 2019-09-12

**Authors:** Cunxian Wang, Chao Hang, Tao Suo, Yulong Li, Pu Xue

**Affiliations:** a School of Aeronautics, Northwestern Polytechnical University, Xi'an, 710072, China; b Fundamental Science on Aircraft Structural Mechanics and Strength Laboratory, Northwestern Polytechnical University, Xi'an, 710072, China; c Commercial Aircraft Corporation of China Ltd, Shanghai, 200126, China

**Keywords:** Ladevèze failure criterion, Quasi-static experiments, Dynamic experiments, Split Hopkinson bar

## Abstract

All parameters of revised Ladevèze failure criterion in Table 1 were determined based on mechanical tests which mainly include quasi-static tensile experiments, quasi-static compressive experiments, quasi-static tensile cyclic loading experiments, the dynamic tensile experiments, dynamic compressive experiments, quasi-static and dynamic inter-laminar shear experiments. The quasi-static experiments were performed using an electronic universal testing machine with a maximum load capacity of 10KN, and the split Hopkinson pressure bar (SHPB) and split Hopkinson tension bar (SHTB) were employed in dynamic experiments. In addition, the parameters of traditional orthogonal anisotropic model and cohesive layer for the laminated plates are listed in Table 2.

**Table:** Specifications Table

Subject	*Engineering*
More specific subject area	*Mechanics of Materials*
Type of data	*Table, Figure*
How data was acquired	*The data are acquired from quasi-static and dynamic mechanical experiments based on electronic universal testing machine CSS-44100, split Hopkinson pressure bar (SHPB) and split Hopkinson tensile bar (SHTB).*
Data format	*Analyzed*
Parameters for data collection	*Quasi-static tensile experiments, quasi-static compressive experiments, quasi-static tensile cyclic loading experiments, the dynamic tensile experiments, dynamic compressive experiments, quasi-static and dynamic inter-laminar shear experiments.*
Description of data collection	*In*Table 1, Table 2*, the Ladevèze constitutive parameters and the mechanical properties of the unidirectional carbon-epoxy material are presented. All these parameters were determined based on mechanical tests by referring to the ASTM standards. These tests mainly include quasi-static experiments and dynamic experiments (some typical experiments are shown in*Fig. 2*). The quasi-static experiments were performed using an electronic universal testing machine, the strains of specimens were collected by strain gauges as well as the loads were collected by load sensor. In addition, the strain-stress curves were obtained based on one-dimensional stress wave theory by using split Hopkinson pressure bar (SHPB) and split Hopkinson tensile bar (SHTB) were employed in dynamic experiments.*
Data source location	*Institution: Northwestern Polytechnical University* *City/Town/Region: Xi'an* *Country: China*
Data accessibility	*All data is available within this article*
Related research article	*Author's name: Cunxian WANG, Tao SUO, Chao HANG, Yulong LI, Pu XUE, Qiong DENG* *Title: Influence of in-plane tensile preloads on impact responses of laminate composite plates* *Journal: International Journal of Mechanical Sciences* *DOI:*https://doi.org/10.1016/j.ijmecsci.2019.105012

**Table:** 

**Value of the Data** • The set of experimental data presented in the article can support the finite element simulations which helps to gain additional insight into the impact responses of composites. • The experimental method to obtain the data was presented. Researchers who are working on impact dynamics can not only use these data to conduct more simulations but also get corresponding data of another material. • Since strain rate effect of composites is considered, simulations with the support of experimental data corresponding to Ladevèze failure criterion can predict the damage mechanisms better than traditional biphasic model under high-velocity impact loading condition, which can significantly reduce research costs.

## 1 Data

**Fig. 1:**
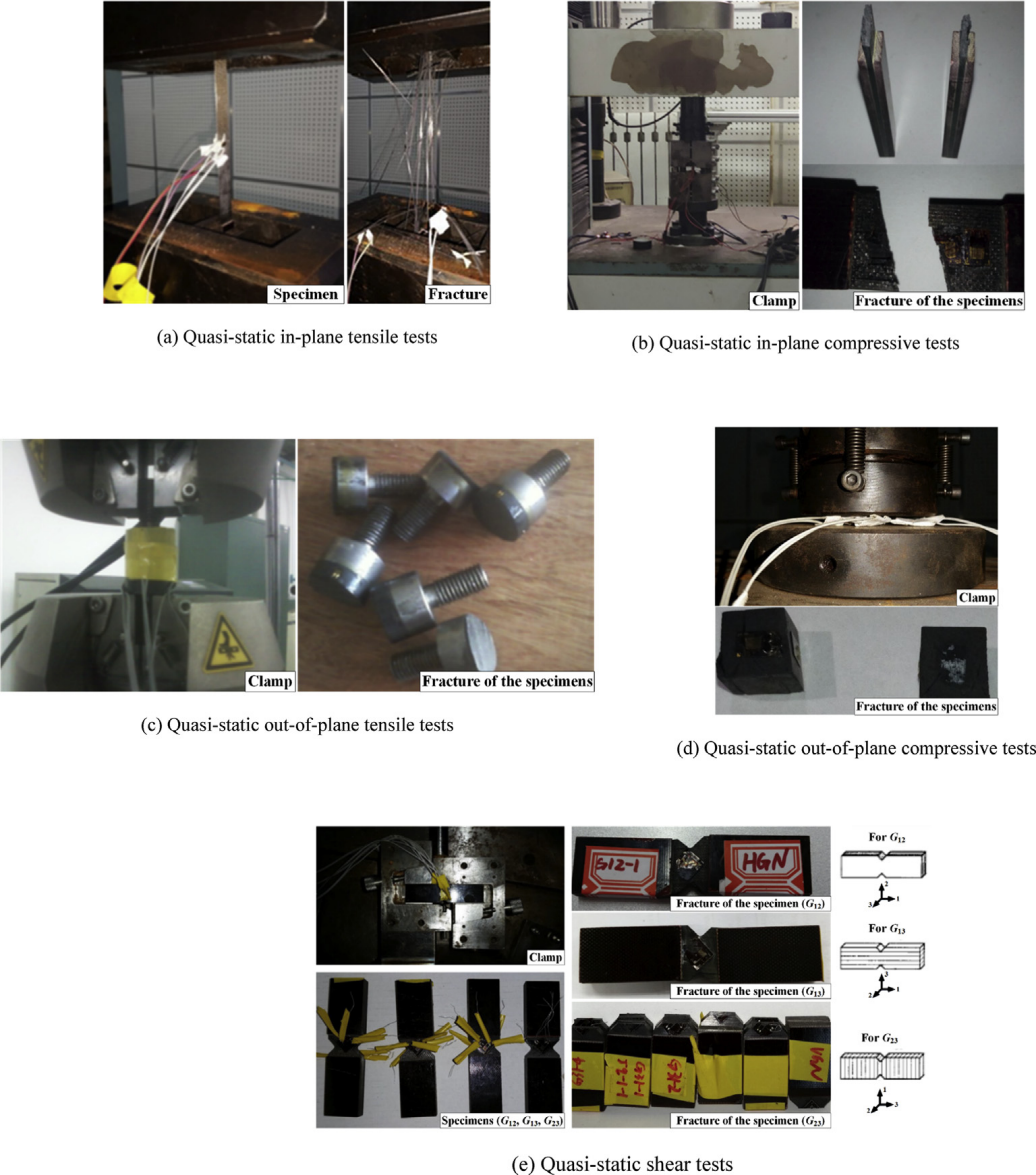
Typical experimental results for determination of mechanical properties of the unidirectional carbon-epoxy material.

**Fig. 2:**
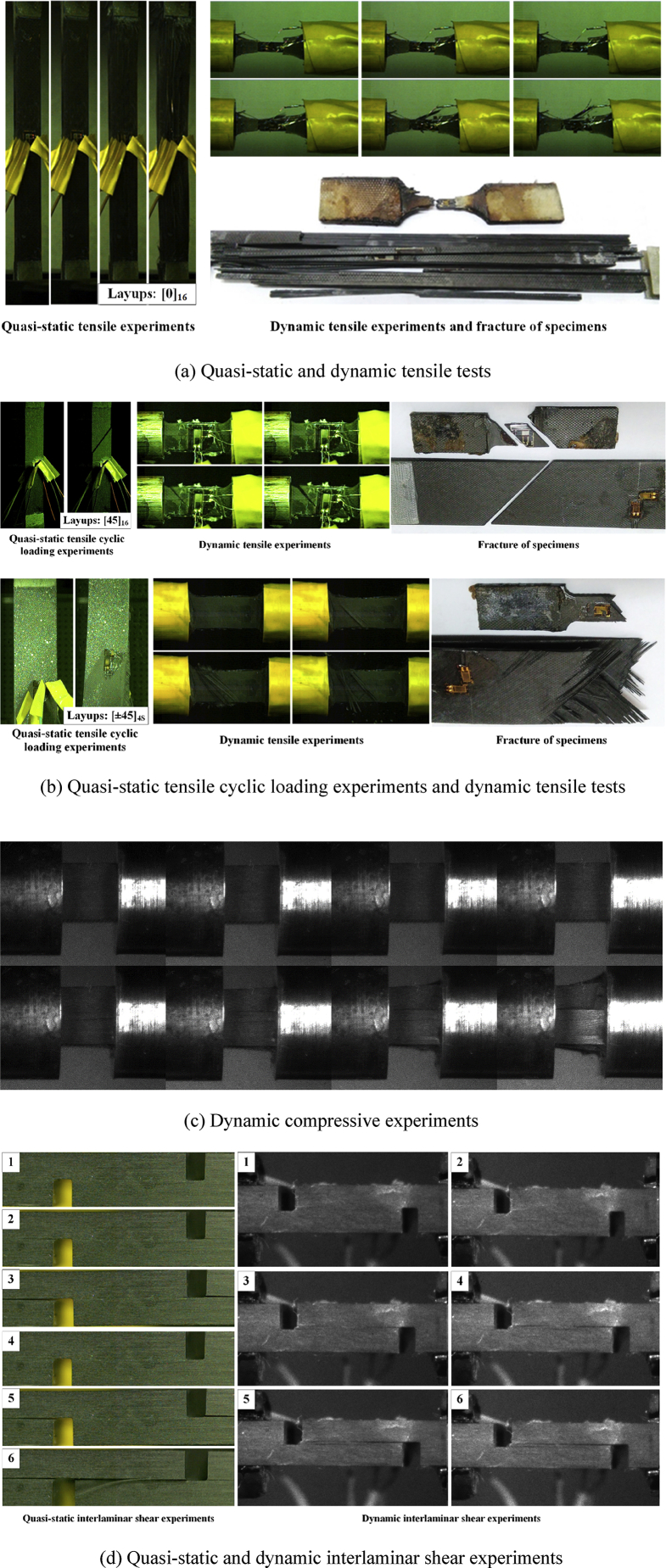
Typical experimental results for determination of Ladevèze constitutive parameters of the unidirectional carbon-epoxy material.

The data presented in this section contains Ladevèze constitutive parameters of unidirectional carbon-epoxy material and orthotropic material properties for composites and cohesive element. All parameters in Table 1 were determined based on mechanical tests which mainly include quasi-static tensile experiment, quasi-static compressive experiment, quasi-static tensile cyclic loading experiment, the dynamic tensile experiment, dynamic compressive experiment, quasi-static and dynamic inter-laminar shear experiment (some typical experiments are shown in Fig. 2). In order to calculate the parameters of cohesive element by using the method mentioned in related original research article [3], the mechanical properties of the unidirectional carbon-epoxy material need to be experimentally obtained, thus several kinds of quasi-static mechanical experiments were performed using an electronic universal testing machine by referring to the ASTM standards (as shown in Fig. 1). The elasticity modulus, Poisson's ratio and fracture strength of composites can be obtained from the quasi-static in-plane and out-of-plane experiments (as shown in Fig. 1(a)–(d)), the shear modulus and shear strength of composites can also be obtained from three kinds of quasi-static shear test which corresponding three shear directions (as shown in Fig. 1(e)).

**Table 1:** Ladevèze constitutive parameters of unidirectional carbon-epoxy material.

	Parameters	Values
Basic elastic parameters	Density, *ρ* (g/cm^3^)	1.51 (g/cm^3^)
	Young's modulus, $E_{1}^{0t}$	151800(MPa)
	Young's modulus, $E_{2}^{0}$	12100(MPa)
	Young's modulus, ${\text{E}}_{1}^{\text{0C}}$	134754MPa
	Shear modulus, $G_{12}^{0}$	3300MPa
	Dynamic shear modulus, G13	3397.50MPa
	Poison's ratio, $v_{12}^{0}$	0.319
	Nonlinear correction factor, γ	1.55E-5
Damage evolution parameters	Horizontal critical damage limit, $Y_{C}^{\prime }$	2.747$\sqrt{MPa}$
	Shear critical damage limit, $Y_{C}$	2.035$\sqrt{MPa}$
	Initial horizontal damage threshold, $Y_{0}^{\prime }$	0.451$\sqrt{MPa}$
	Initial shear damage threshold, $Y_{0}$	0.397$\sqrt{MPa}$
	Brittle-damage threshold, $Y_{s}^{\prime }$	0.858$\sqrt{MPa}$
	Damage limit, $Y_{R}$	1.745$\sqrt{MPa}$
	Coupling coefficient of horizontal damage and shear damage, b	1.754
	Longitudinal tensile strain threshold, $\varepsilon_{\text{i}}^{\text{ft}}$	0.0106
	Longitudinal tensile strain limit, $\varepsilon_{\text{u}}^{\text{ft}}$	0.0106
	Longitudinal tensile and damage, ${\text{d}}_{\text{u}}^{\text{ft}}$	0.99
	Longitudinal compressive damage, ${\text{d}}_{\text{u}}^{\text{fc}}$	0.99
	Longitudinal compressive strain threshold,$\varepsilon_{\text{i}}^{\text{fc}}$	0.011
	Longitudinal compressive strain limit, $\varepsilon_{\text{u}}^{\text{fc}}$	0.011
Plastic parameters	Initial yield stress, $R_{0}$	25.1MPa
	Hardening coefficient, β	221.905
	Hardening index, m	0.247
	Coupling coefficient of horizontal strain and shear strain, $a^{2}=A$	0.33
Fixed parameter for longitudinal elastic modulus	${\dot{\varepsilon }}_{11}^{\text{ref}}$	0.0003
	$D_{11}$	0.0256
	$n_{11}$	−0.3255
Fixed parameter for longitudinal fracture strain	${\dot{\varepsilon }}_{11}^{\text{ref}}$	0.0003
	$D_{11}^{u}$	−0.018
	$n_{11}^{u}$	0.3385
Fixed parameter for horizontal elastic modulus	${\dot{\varepsilon }}_{22}^{\text{ref}}$	0.0003
	$D_{22}$	0.0727
	$n_{22}$	−0.92289
Fixed parameter for shear modulus	${\dot{\varepsilon }}_{12}^{\text{ref}}$	0.0003
	$D_{12}$	0.0329
	$n_{12}$	−0.4208
Fixed parameter for yield stress	${\dot{\varepsilon }}_{12}^{\text{ref}}$	0.0003
	$D_{R0}$	0.8615
	$n_{R0}$	−1.8721

**Table 2:** Orthotropic material properties for composites and cohesive element

Parameters	Values	Parameters	Values
Density, *ρ*	1.51 (g/cm^3^)	*X*_*t*_	1872 (MPa)
*E*_11_	151.8 (GPa)	*X*_*c*_	776 (MPa)
*E*_22_	12 (GPa)	*Y*_*t*_	34 (MPa)
*E*_33_	12 (GPa)	*Y*_*c*_	150 (MPa)
*ν*_12_	0.3	*K*_*nn*_	4.8 × 10^6^(N/mm^3^)
*ν*_13_	0.3	*K*_*ss*_	2.64 × 10^6^(N/mm^3^)
*ν*_23_	0.38	*K*_*tt*_	2.64 × 10^6^(N/mm^3^)
*G*_12_	3.3 (GPa)	*σ*_*n*_	34 (MPa)
*G*_13_	3.3 (GPa)	*σ*_*s*_	100 (MPa)
*G*_23_	3.3 (GPa)	*σ*_*t*_	100 (MPa)
*S*_12_	100 (MPa)	*G*_*IC*_	600 (J/m^2^)
*S*_13_	100 (MPa)	*G*_*IIC*_	1200 (J/m^2^)
*S*_23_	100 (MPa)	*G*_*IIIC*_	1200 (J/m^2^)

where: “1” represents the fiber direction; “2” represents the transverse direction; “3” represents the through-thickness direction. Some typical experimental results are shown in Fig. 1.

## 2 Experimental design, materials, and methods

For Ladevèze failure criterion, it is considered to be a failure mode which can analyze the damage and failure of the composite laminated plate. The damage kinematics of the elementary layer of Ladevèze failure criterion can be depicted as follows [2]:

Global constitutive relation:

(A.1) $$\left\{\begin{array}{c} \varepsilon_{11}^{e}\\ \varepsilon_{22}^{e}\\ 2\varepsilon_{12}^{e}\\ 2\varepsilon_{23}^{e}\\ 2\varepsilon_{13}^{e} \end{array}\right\}=\left[\begin{array}{ccccc} \frac{1}{E_{1}} & -\frac{v_{12}^{0}}{E_{1}} & 0 & 0 & 0\\ -\frac{v_{12}^{0}}{E_{1}} & \frac{1}{E_{2}} & 0 & 0 & 0\\ 0 & 0 & \frac{1}{G_{12}} & 0 & 0\\ 0 & 0 & 0 & \frac{1}{G_{23}^{0}} & 0\\ 0 & 0 & 0 & 0 & \frac{1}{G_{13}^{0}} \end{array}\right]\left\{\begin{array}{l} \sigma_{11}\\ \sigma_{22}\\ \sigma_{12}\\ \sigma_{23}\\ \sigma_{13} \end{array}\right\}$$

(a) Longitudinal (direction 1, in the fiber direction):

For tension, the damage evolution can be depicted as:$E_{1}=E_{1}^{t}$, ($\varepsilon_{11}>0$); Subcritical state: $E_{1}^{t}=E_{1}^{0t}$, if $0<\varepsilon_{11}<\varepsilon_{i}^{ft}$; Critical state: $E_{1}^{t}=E_{1}^{0t}\left(1-d^{ft}\right)$, $d^{ft}=d_{u}^{ft}\frac{\varepsilon_{11}-\varepsilon_{i}^{ft}}{\varepsilon_{u}^{ft}-\varepsilon_{i}^{ft}}$, if $\varepsilon_{i}^{ft}\leq \varepsilon_{11}<\varepsilon_{u}^{ft}$; Beyond the critical state: $E_{1}^{t}=E_{1}^{0t}\left(1-d^{ft}\right)$, $d^{ft}=1-\left(1-d_{u}^{ft}\right)\frac{\varepsilon_{u}^{ft}}{\varepsilon_{11}}$, if $\varepsilon_{u}^{ft}\leq \varepsilon_{11}<\infty$; In these equations, $E_{1}^{t}$ are the longitudinal tensile modulus of elasticity, $E_{1}^{0t}$ is the initial longitudinal tensile elastic modulus of elasticity, $d^{ft}$ denotes the longitudinal tensile damage factor.

For compression, the damage evolution can be depicted as follows:$E_{1}=E_{1}^{c}$, if $\varepsilon_{11}<0$, $E_{1}^{\gamma }=E_{1}^{0c}/\left(1+\gamma E_{1}^{0c}\left|\varepsilon_{11}\right|\right)$, where $E_{1}^{0c}$ is the initial compressive modulus of elasticity; Subcritical state:$E_{1}^{c}=E_{1}^{\gamma }$, if $\left|\varepsilon_{11}\right|<\varepsilon_{i}^{fc}$; Critical state:$E_{1}^{c}=E_{1}^{\gamma }\left(1-d^{fc}\right)$, $d^{fc}=d_{u}^{fc}\frac{\left|\varepsilon_{11}\right|-\varepsilon_{i}^{fc}}{\varepsilon_{u}^{fc}-\varepsilon_{i}^{fc}}$, if $\varepsilon_{i}^{fc}\leq \left|\varepsilon_{11}\right|<\varepsilon_{u}^{fc}$; When beyond the critical state:$E_{1}^{c}=E_{1}^{\gamma }\left(1-d^{fc}\right)$, $d^{fc}=1-\left(1-d_{u}^{fc}\right)\frac{\varepsilon_{u}^{fc}}{\left|\varepsilon_{11}\right|}$, if $\varepsilon_{u}^{fc}\leq \left|\varepsilon_{11}\right|<\infty$, where $E_{1}^{c}$ are the longitudinal compressive modulus of elasticity, $E_{1}^{0t}$ is the initial longitudinal compressive modulus of elasticity, $E_{1}^{\gamma }$is the longitudinal nonlinear compressive modulus of elasticity, γ is the nonlinear correction factor of $E_{1}^{\gamma }$, $d^{fc}$ denotes the longitudinal compressive damage factor.

(b) Horizontal (direction 2, in the transverse direction):

(A.2) $$E_{2}=E_{2}^{0}\left(1-d^{\prime }\right),\text{if}\;\varepsilon_{22}>0\;\text{otherwise}\;E_{2}=E_{2}^{0}$$

where $E_{2}^{0}$ is the initial value of $E_{2}$, $d^{\prime }$ is equivalent to take the transverse modulus as a scalar-damage variable that remain constant throughout the ply thickness.

(c) Normal (direction 3, in the through-thickness direction)

(A.3) $$G_{12}=G_{12}^{0}\left(1-d\right)$$

where $G_{12}^{0}$ is the initial value of $G_{12}$, d is equivalent to take the shear modulus as a scalar-damage variable that remain constant throughout the ply thickness. $G_{23}^{0}$ is the same as $G_{23}$.

From the direction 2 and direction 3, the damage parameter $d^{\prime }$ is applied to depict the debonding of fiber/matrix, d is used to depict the microcracking of the matrix. Therefore, two conjugate quantities are defined by Ladevèze [1] associated with damage variables as follows:

(A.4) $$\frac{\partial E_{D}}{\partial d^{\prime }}=Y_{d}^{\prime }=\frac{1}{2}\frac{{\langle \sigma_{22}^{2}\rangle }_{+}}{E_{2}^{0}{\left(1-d^{\prime }\right)}^{2}}$$

(A.5) $$\frac{\partial E_{D}}{\partial d}=Y_{d}=\frac{1}{2}\frac{\sigma_{12}^{2}+\sigma_{13}^{2}}{G_{12}^{0}{\left(1-d\right)}^{2}}$$

In addition, the damage-time functions are defined by Ladevèze as:

(A.6) $$Y\left(t\right)=Sup_{\tau \leq t}\sqrt{Y_{d}\left(\tau \right)+bY_{d}^{\prime }\left(\tau \right)}$$

(A.7) $$Y^{\prime }\left(t\right)=Sup_{\tau \leq t}\sqrt{Y_{d}^{\prime }\left(\tau \right)}$$

Based on Ladevèze damage model, the damage-development laws can be defined as:

(A.8) $$d=\left\{ \begin{array}{l} 0\;\;\;\;\;Y\left(t\right)<Y_{0}\\ \frac{{\langle Y\left(t\right)-Y_{0}\rangle }_{+}}{Y_{c}^{\prime }}\;d<d_{\max},Y\left(t\right)<Y_{R},Y^{\prime }\left(t\right)<Y_{s}\\ d_{\max}\;\;\;\;Y\left(t\right)>Y_{0},d\geq d_{\max},Y\left(t\right)\geq Y_{R},Y^{\prime }\left(t\right)\geq Y_{s} \end{array}\right.$$

(A.9) $$d^{\prime }=\left\{ \begin{array}{l} 0\;\;\;\;\;Y\left(t\right)<Y_{0}^{\prime }\\ \frac{{\langle Y\left(t\right)-Y_{0}^{\prime }\rangle }_{+}}{Y_{c}^{\prime }}\;d^{\prime }<d_{\max},Y\left(t\right)<Y_{R},Y^{\prime }\left(t\right)<Y_{s}\\ d_{\max}\begin{array}{cccc} & & & Y\left(t\right)>Y_{0}^{\prime },d^{\prime }\geq d_{\max},Y\left(t\right)\geq Y_{R},Y^{\prime }\left(t\right)\geq Y_{s} \end{array} \end{array}\right.$$

where $Y_{c}^{\prime }$ and $Y_{c}$ denote the horizontal critical damage limit and the shear critical damage limit. $Y_{0}^{\prime }$ is the initial horizontal damage threshold, $Y_{0}$ is the initial shear damage threshold, $Y_{R}^{\prime }$ is the brittle-damage threshold, the new parameter $Y_{R}$ is defined as a damage limit to ensure the multiple loop computations, $Y_{S}$ is the brittle-damage threshold which determines the behavior of the fiber/matrix interface in transverse tension. In this paper, the *d*_max_ in the VUMAT is defined as 0.99 instead of 1, and the multiple loop computations can be finished by two judgments, one is the $d_{n}-d_{n-1}\leq 1\times 10^{-5}$ or $d_{n}^{'}-d_{n-1}^{'}\leq 1\times 10^{-5}$, the other one is $d>d_{\max}$ or $d^{\prime }>d_{\max}$.

As known, the organic matrix always shows strain rate sensitivity and also appears the viscous state under high-speed impact. The composites under different strain rates showed that the strain rate sensitivity barely improves under the lower strain rate but increases obviously when reaching a high value of strain rate. Therefore, the viscous stresses can be applied to revise the effect of strain rate. As known, the constitutive relationship can be depicted as

(A.10) $$\left\{\begin{array}{c} \sigma_{11}\\ \sigma_{22}\\ \sigma_{12}\\ \sigma_{13}\\ \sigma_{23} \end{array}\right\}=\left(\begin{array}{ccccc} C_{11} & \nu_{21}^{0}C_{11} & 0 & 0 & 0\\ \nu_{21}^{0}C_{11} & C_{22}\left(1-d^{\prime }\right) & 0 & 0 & 0\\ 0 & 0 & C_{12}\left(1-d\right) & 0 & 0\\ 0 & 0 & 0 & G_{13}^{0} & 0\\ 0 & 0 & 0 & 0 & G_{23}^{0} \end{array}\right)\left\{\begin{array}{c} \varepsilon_{11}^{e}\\ \varepsilon_{22}^{e}\\ 2\varepsilon_{12}^{e}\\ 2\varepsilon_{13}^{e}\\ 2\varepsilon_{23}^{e} \end{array}\right\}$$

with

(A.11) $${\text{C}}_{\text{ij}}={\left({\text{C}}_{\text{ij}}^{\text{0}}\right)\left(1+{\text{F}}_{\text{ij}}\text{(}\dot{\text{ε}}\text{)}\right)|}_{\text{i,j}=1\text{,}2},{\text{C}}_{\text{ij}}^{\text{0}}={{\text{E}}_{\text{i}}^{\text{0}}|}_{\text{i}=\text{j}}\;or\;{\text{C}}_{\text{ij}}^{\text{0}}={{\text{G}}_{\text{ij}}^{\text{0}}|}_{\text{i}\neq \text{j}}$$

The function needs to be defined by three parameters (${\dot{\text{ε}}}_{\text{ij}}^{\text{ref}}$, ${\text{D}}_{\text{ij}}$, ${\text{n}}_{\text{ij}}$). In the VUMAT, the logarithmic and linear relationship are chosen based on the dynamic experimental results of [0]_16_, [±45]_4S_ and [45]_16_ specimens. The relationship can be defined as follows:

Logarithmic:

(A.12) $$F_{ij}\left(\dot{\varepsilon }\right)=D_{ij}\;\log\left(\frac{\dot{\varepsilon }}{{\dot{\varepsilon }}_{ij}^{ref}}\right)+\log\left(n_{ij}\right)$$

Linear:

(A.13) $$F_{ij}\left(\dot{\varepsilon }\right)=D_{ij}\left(\frac{\dot{\varepsilon }}{{\dot{\varepsilon }}_{ij}^{ref}}\right)+n_{ij}$$

Table 3 presents the experiments design for revised Ladevèze failure criterion, some typical experimental results are shown in Fig. 2.

**Table 3:** Experiments design for revised Ladevèze failure criterion.

Experiment type	Layups	Length	Width	Height	Number
quasi-static tensile experiment	[0]_16_	250mm	15 mm	2 mm	14
quasi-static tensile cyclic loading experiment	[0]_16_	250 mm	15 mm	2 mm	14
	[45]_16_	250 mm	25 mm	2 mm	14
	[±67.5]_4S_	250 mm	25 mm	2 mm	14
	[±45]_4S_	250 mm	25 mm	2 mm	14
quasi-static compressive experiment	[0]_16_	140 mm	12 mm	2 mm	14
dynamic tensile experiment	[0]_16_	75 mm	14 mm	2 mm	21
	[±45]_4S_	75 mm	14 mm	2 mm	21
	[45]_16_	75 mm	14 mm	2 mm	21
dynamic compressive experiment	[0]_48_	6 mm	6 mm	6 mm	21
quasi-static interlaminar shear experiment	[0]_48_	14 mm	6 mm	6 mm	14
dynamic interlaminar shear experiment	[0]_48_	14 mm	6 mm	6 mm	14
